# Characterization of volatile thiols in Chinese liquor (Baijiu) by ultraperformance liquid chromatography–mass spectrometry and ultraperformance liquid chromatography–quadrupole-time-of-flight mass spectrometry

**DOI:** 10.3389/fnut.2022.1022600

**Published:** 2022-10-03

**Authors:** Yan Yan, Jun Lu, Yao Nie, Changwen Li, Shuang Chen, Yan Xu

**Affiliations:** ^1^School of Liquor and Food Engineering, Guizhou University, Guiyang, China; ^2^Laboratory of Brewing Microbiology and Applied Enzymology, Key Laboratory of Industrial Biotechnology of Ministry of Education, School of Biotechnology, Jiangnan University, Wuxi, China; ^3^Guizhou Guotai Liquor Group Co., Ltd., Renhuai, China

**Keywords:** thiols, Baijiu, UPLC-MS/MS, quantification, odor activity value

## Abstract

Volatile thiols give a unique flavor to foods and they have been extensively studied due to their effects on sensory properties. The analytical assay of volatile thiols in food is hindered by the complexity of the matrix, and by both their high reactivity and their typically low concentrations. A new ultraperformance liquid chromatography (UPLC) strategy has been developed for the identification and quantification of volatile thiols in Chinese liquor (Baijiu). 4,4’-Dithiodipyridine reacted rapidly with eight known thiols to form derivatives, which provided a diagnostic fragment ion (*m/z* 143.5) for tandem mass spectrometry (MS/MS). To screen for new thiols, Baijiu samples were analyzed by means of UPLC–MS/MS screening for compounds exhibiting the diagnostic fragment ion (*m/z* X→143.5). New peaks with precursor ions of *m/z* 244, 200 and 214 were detected. Using UPLC with quadrupole-time-of-flight mass spectrometry (UPLC–Q-TOF–MS) and authentic standards, ethyl 2-mercaptoacetate, 1-butanethiol, and 1-pentanethiol were identified in Baijiu for the first time. Commercial Baijiu samples were analyzed with the new method and the distribution of 11 thiols was revealed in different Baijiu aroma-types. The aroma contribution of these thiols was evaluated by their odoractivity values (OAVs), with the result that 7 of 11 volatile thiols had OAVs > 1. In particular, methanethiol, 2-furfurylthiol, and 2-methyl-3-furanthiol had relatively high OAVs, indicating that they contribute significantly to the aroma profile of Baijiu.

## Introduction

Baijiu (Chinese liquor) is a locally-produced, distilled alcoholic beverage that has been very popular for thousands of years and is produced using a unique traditional solid-state fermentation process (1, 2). It is typically made from sorghum or a mixture of wheat, barley, corn, rice, and sorghum. Baijiu is produced using traditional spontaneous fermentation processes with an assortment of microbial communities involved (3). The characteristic aroma of Baijiu can vary considerably, resulting from differences in raw materials, production processes, and flavor components. Baijiu is generally classified into 12 aroma types (4). At present, soy sauce aroma-type Baijiu (SSAB) (5, 6), strong aroma-type Baijiu (SAB) (7, 8), light aroma-type Baijiu (LAB) (9, 10), and roasted sesame-like aroma-type Baijiu (RSAB) (11, 12) are the four common Baijiu aroma-types in China.

The popularity of Baijiu arises mainly from its pleasant taste and the odor active compounds in its volatile fraction. More than 1,870 volatile compounds have been identified in Baijiu, including esters, alcohols, ketones, acids, aldehydes, nitrogenous compounds, and sulfur compounds (4). Despite this great complexity, only a small number of compounds are responsible for the majority of the olfactory sensation Baijiu provides. Some sulfur containing compounds are among the most important for Baijiu flavor (13, 14). In particular, volatile thiols, known historically as mercaptans, have the general structure, R-SH, and exhibit important sensory effects to Baijiu, because their concentrations are much higher than their low odor thresholds (12, 15, 16). Therefore, their determination and insights into their concentrations could help to improve the sensory quality of Baijiu and modulate its sensory attributes.

Several methods for analyzing volatile thiols in Baijiu have been developed, all using gas chromatography (GC) (12, 17). However, profiling volatile thiols in Baijiu remains a bottleneck with GC-based methods. The complexity of the Baijiu sample matrix, the typically low concentrations of volatile thiols and their low detection-sensitivity in electron-impact mass spectrometry (EIMS), means that few of them can be identified using GC–MS. Only one volatile thiol was detected in Baijiu by HS-SPME–GC–MS (18). Although pulsed flame photometric detection (PFPD) and sulfur chemiluminescence detection (SCD) are highly selective and sensitive for the quantification of volatile thiols, they provide little identification information, except for their chromatographic retention time. As a result, additional and cumbersome identification procedures are required. Only three volatile thiols were identified by HS-SPME–GC–PFPD, and four volatile thiols were identified by GC × GC–SCD in Baijiu (13, 16). In addition, current GC-based methods for Baijiu volatiles are in general, laborious and time consuming, and some of them involve multiple sample manipulation steps, during which volatile thiols can be lost, or degraded. These limitations have led to the need for a simple, rapid method that enables the identification and quantification of Baijiu volatile thiols.

To improve the sensitivity and selectivity of the measurement method, and stabilize the sulfanyl, thiols from wine and coffee are usually derivatized prior to separation and analysis using liquid chromatography-electrospray ionization mass spectrometry (LC–ESI-MS) (19, 20). 4,4’-Dithiodipyridine (DTDP) is one of the available derivatization reagents. Thiols derivatized with DTDP show increased hydrophobicity, decreased polarity volatility, and stronger affinity for protonation, resulting in an enhanced LC signal for separation and positive-mode ESI-MS detection, which enables the quantification of thiols at ng/L levels in wine (21, 22).

As a result of the low odor thresholds of most volatile thiols, in combination with their low concentrations, it is likely that some potent volatile thiols remain to be discovered in Baijiu. Therefore, we developed a method to identify the volatile thiols by UPLC–MS/MS and UPLC–Q-TOF–MS, rather than adopting a conventional GC approach. The UPLC–MS/MS method was applied to a range of Baijiu samples to investigate the volatile thiol profile.

## Materials and methods

### Samples

Four aroma-types Baijiu samples were under investigation: 8 SSAB samples, 6 SAB samples, 6 RSAB samples, and 7 LAB samples. The detailed information is given in Supplementary Table 1. The samples were stored at room temperature and without light before analysis.

### Chemicals and materials

The thiols studied were methanethiol, ethanethiol, ethyl 2-mercaptoacetate, 2-furfurylthiol, 2-sulfanylethanol, 2-methyl-3-furanthiol, benzenemethanethiol, 3-mercaptohexyl acetate, ethyl 2-mercaptopropionate, 1-butanethiol, 1-pentanethiol. The internal standard was 2-phenylethanethiol. All analytes were provided commercially at high-purity grade (>°96%) by Sigma-Aldrich (Shanghai, China). Ethylenediaminetetraacetic acid disodium salt (EDTA-Na_2_), 4,4’-Dithiodipyridine (DTDP, 97%), acetaldehyde (99%), and formic acid (99%) were bought from J&K Chemical Corp., (Beijing, China). C18 solid-phase extraction cartridges (6 mL, 500 mg) were purchased from ANPEL (Shanghai, China). LC–MS grade acetonitrile were purchased from Merck (Sigma-Aldrich, Shanghai, China).

### Sample preparation and derivatization

A modified derivatization method for the analysis of thiols in Baijiu was used according to a previously described procedure (21). A solution of the Baijiu sample (20 mL) was spiked with 10 μL of 2-phenylethanethiol (6 mg/L) and used as an internal standard solution. The sample was diluted with water (20 mL, Millipore, USA) to a final concentration of about 25% ethanol by volume. EDTA-Na_2_ (40 mg), 50% acetaldehyde (160 μL), and freshly thawed DTDP (10 mM, 400 μL) was then added to the resulting solution. The mixture was vortex-assisted stirred for 5 min and rested for 25 min at room temperature. The sample was loaded onto a SPE cartridge, which was previously pretreated with 6 mL of methanol, followed by 6 mL of water. The column was washed with 50% methanol (12 mL). The analytes retained by SPE were eluted with methanol (3 mL) and concentrated to a final volume of 400 μL under nitrogen. The solution was filtered (0.22 μm) and stored at 4°C.

### Mass spectrometry method development

A triple-quadrupole mass spectrometer (Xevo TQ-S, Waters, Milford, USA) was performed with positive ionization mode. The multiple-reaction-monitoring (MRM) conditions were optimized with infusion of derivatized thiols at 10 μL/min. Based on the mass spectra (Supplementary Figure 1) with the MassLynx software using the sample tune and develop method, two ion transitions were chosen for the quantification (quantifier) and the confirmation (qualifier) (23). Table 1 lists the best MRM parameters for each derived thiol with the following instrument settings: capillary voltage, 3 kV; desolvation temperature, 500°C; gas, 800 L/h.

**TABLE 1:** Optimized multiple-reaction-monitoring (MRM) parameters for the derivatizeds thiol in Baijiu.

No.	Compounds	Retention time	Precursor ion	Product ion	Cone voltage	Collision energy	Type
1	Methanethiol	1.45	158.0	110.7	23	19	Quantifier
			158.0	143.1	23	20	Qualifier
2	Ethanethiol	2.46	172.0	143.0	23	19	Quantifier
			172.0	110.2	24	21	Qualifier
3	Ethyl 2-mercaptoacetate	2.66	230.0	143.4	25	27	Quantifier
			230.0	201.1	27	29	Qualifier
4	2-Furfurylthiol	4.06	223.7	143.8	21	19	Quantifier
			223.7	81.3	24	21	Qualifier
5	2-Sulfanylethanol	6.95	187.6	173.0	21	15	Quantifier
			187.6	143.6	22	17	Qualifier
6	2-Methyl-3-furanthiol	8.94	224.0	110.6	23	25	Quantifier
			224.0	143.8	23	23	Qualifier
7	Benzenemethanethiol	9.03	234.0	143.9	23	17	Quantifier
			234.0	110.5	25	23	Qualifier
8	3-Mercaptohexyl acetate	15.59	286.2	143.3	23	23	Quantifier
			286.2	81.3	24	25	Qualifier
9	Ethyl 2-mercaptopropionate	4.97	244.0	143.2	25	27	Quantifier
			244.0	110.2	27	29	Qualifier
10	1-Butanethiol	10.78	199.7	143.4	25	19	Quantifier
			199.7	110.7	25	28	Qualifier
11	1-Pentanethiol	16.39	214.0	143.2	27	17	Quantifier
			214.0	110.5	27	19	Qualifier

### Ultraperformance liquid chromatography–mass spectrometry instrumentation and conditions

The thiols were separated by using an UPLC system (Waters, Milford, USA) equipped with a vacuum degasser, a binary solvent manager, and an autosampler. As stationary phase an analytical column (waters BEH C18, 100 × 2.1 mm, 1.7 μm) was used. Flow-rate was 0.3 mL/min and the composition of eluents was: solvent A (0.1% formic acid in water) and solvent B (0.1% formic acid in acetonitrile). The linear gradient for solvent B was as follows: 0 min, 15%; 13 min, 22%; 14 min, 30%; 18 min, 35%; 18.5 min, 100%; 21.5 min, 100%; and 22 min, 15%. The injection volume was set at 10 μL.

The derivatization Baijiu sample was analyzed by means of UPLC–MS/MS using precursor ion scan screening for compounds releasing the diagnostic ion (*m/z* X→143.5) (24). In the source, cone voltage of 23 V and collision energy of 20 eV was applied. The eleven derivatives were quantified in MRM mode by monitoring their corresponding precursor ion, product ion, cone voltage, and collision energy, respectively (Table 1). Each Baijiu sample was tested in three different sessions to obtain an average value.

### Thiols identification by ultraperformance liquid chromatography–quadrupole-time-of-flight mass spectrometry

The derivatized Baijiu sample was carried out by UPLC system (Waters, Milford, USA), coupled to a SYNAPT Q–TOF mass spectrometer (Waters, Milford, USA), using the following operation parameters: capillary voltage: 3,500 V; cone voltage: 20 V; collision energy: 20 eV; source temperature: 100°C; desolvation temperature: 400°C; desolvation gas flow: 700 L/h; cone gas flow: 50 L/h. The analytical column, mobile phase, and linear gradient were the same as those in the UPLC–MS/MS. The precursor ions were selected from UPLC–MS/MS spectra, which were produced by precursor ion scan mode. Then, the target ions were fragmented by collision-induced dissociation.

### Method validation

Thiols standard solutions at different concentrations were obtained by diluting their corresponding stock solutions using a 50% ethanol-water solution and derivatized *via* the workflow developed in this study. The linearity of the derivatized thiol was evaluated according to the relative peak area versus the concentration and expressed using the correlation coefficient (*R*^2^). Relative to the calibration curve, thiol standards were added to the Baijiu samples at low, medium and high concentration levels. The recovery was calculated based on the concentrations of thiols measured in the spiked and non-spiked Baijiu samples. The intra-day precision was evaluated using the repeated analysis of 11 thiols found in the same Baijiu sample five times on the same day and the inter-day precision was determined on five consecutive days. The limits of detection (LOD) and limits of quantitation (LOQ) of the thiols were determined at their concentrations when the signal/noise (S/N) ratio was 3 and 10, respectively (25).

### Determination of odor thresholds

Using a previously proposed method (26), the thiol odor thresholds were detected in a 46% ethanol-water solution using three-alternative forced choice tests. A sensory panel consisting of 20 panelists, 10 males, and 10 females, with an average age of 25 years. The odor activity value (OAV) was defined by dividing the concentration of the thiol to its odor threshold (27, 28).

### Statistical analyses

The Masslynx software (waters) was used to process the data of UPLC–MS/MS and UPLC–Q-TOF–MS. Statistical analyses were carried out by using the Microsoft Excel 2010. Principal component analysis (PCA) of the concentrations of thiols in four different aroma-types Baijiu by XLSTAT 2018 software. Variable importance for projection (VIP) values were carried out using the SIMCA software.

## Results and discussion

### Identification of new thiols in Baijiu by ultraperformance liquid chromatography–mass spectrometry and ultraperformance liquid chromatography–quadrupole-time-of-flight mass spectrometry

#### Ultraperformance liquid chromatography–mass spectrometry analysis of derivatized thiols using multiple-reaction-monitoring

**FIGURE 1:**
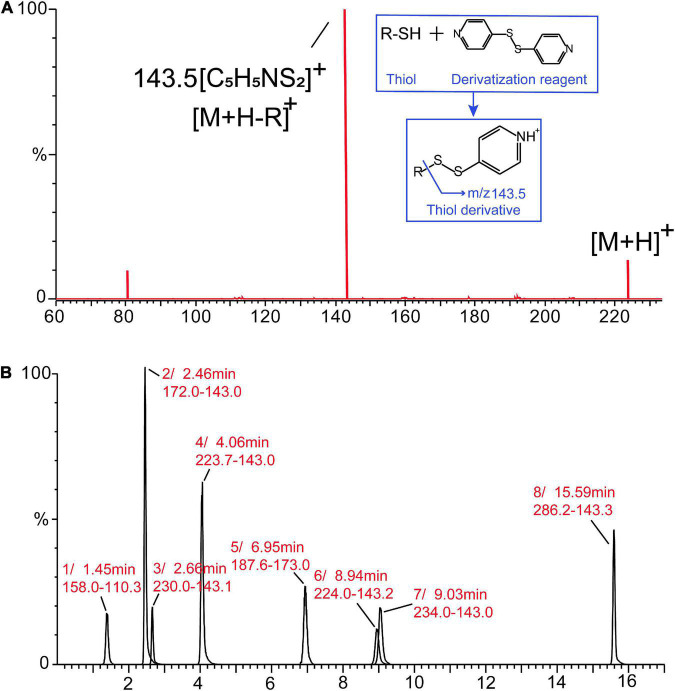
Mass spectrometry (MS/MS) spectrum of the derivatized thiols **(A)**. Multiple-reaction-monitoring (MRM) analysis of the derivatized thiol standards (1–8) in positive mode by ultraperformance liquid chromatography (UPLC)–mass spectrometry (MS/MS) **(B)**.

For the development of the MRM method, each commercial thiol standard derivative (1–8) was directly infused into the MS ion source in positive mode. The cone voltage (1–50 V) and collision energy (1–35 eV) were optimized to obtain the precursor ion and product ion with maximum fragment ion abundance. Table 1 lists the optimal MRM parameters for each thiol derivative based on their response and ion fragmentation (Supplementary Figure 1). All the derivatized thiols have a diagnostic ion observed between *m/z* 143 and 144 corresponding to a disulfanylpyridine group arising from the derivatized portion of the precursor ion. The fragmentation pathways and ions were similar to those previously reported (21); the fragment ion observed at *m/z* 143.5 in this study was used as the diagnostic ion for identification of the derivatized thiols (Figure 1A). To obtain a good separation of the derivatized thiols, several parameters in the chromatography method were systematically varied, allowing the derivatized thiols to be separated within 17 min, using the optimized gradient elution procedure (Figure 1B).

#### Precursor ion scan of *m/z* 143.5 in Baijiu by ultraperformance liquid chromatography–mass spectrometry

**FIGURE 2:**
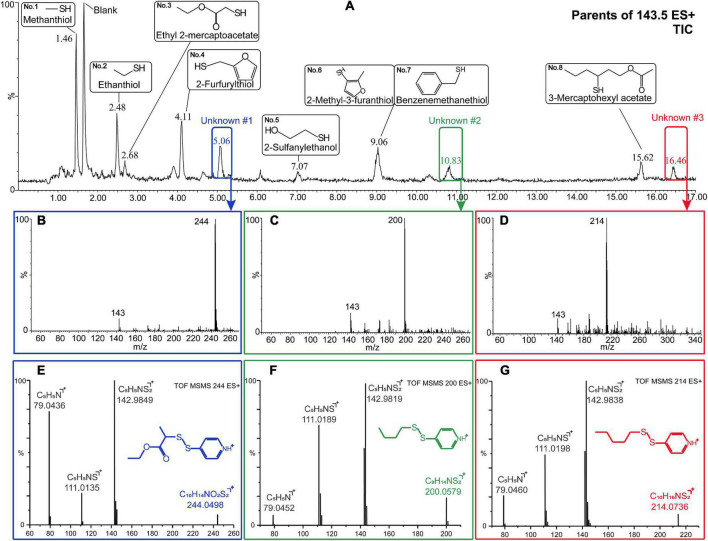
Analysis of the derivatized Baijiu sample using precursor mode and ultraperformance liquid chromatography (UPLC)–mass spectrometry (MS/MS) screening for compounds releasing a disulfanylpyridin ion (*m/z* X→143.5) **(A)**. MS spectra of the three unknown peaks by UPLC–MS/MS **(B–D)**. MS peaks observed at 5.06 (unknown #1), 10.83 (unknown #2), and 16.46 min (unknown #3) using ultraperformance liquid chromatography quadrupole-time-of-flight mass spectrometry (UPLC–Q-TOF–MS) and their corresponding elemental composition **(E–G)**.

Based on the fragmentation behavior mentioned above, the *m/z* 143.5 peak was chosen as the diagnostic ion for the derivatized thiols. To improve the detection sensitivity of the constituent of interest, the volume of the SSAB sample was increased to 120 mL, to obtained a higher concentration of derivatized thiols. Based on the cone voltage and collision energy of the derivatized thiols (1–8), a cone voltage of 23 V and collision energy of 20 eV, were found to maximize the fragment ion abundance of the peak between *m/z* 143 and 144, for most of the derivatized thiols. Under these conditions, precursor ion scanning of *m/z* 143.5 was applied, to produce a single-ion chromatogram (Figure 2A). Following the diagnostic ion screening (*m/z* X→143.5), the peaks corresponding to the derivatized thiol candidates were exposed from the total ion chromatogram. Eight peaks (1–8) were identified as known thiols in Baijiu, by comparing the chromatographic retention time (Figure 1B) and MS fragmentation, with the authentic standards. Other than the eight known peaks, Figure 2A also shows three new peaks, unknown #1 (5.06 min), unknown #2 (10.83 min), and unknown #3 (16.46 min) in the chromatogram; three precursor ions at *m/z* 244, 200 and 214 were extracted from the three peaks, respectively (Figures 2B–D). The three peaks appeared to be potential new thiols, so their structures were identified using the following UPLC–Q-TOF–MS method.

#### New thiols in Baijiu

Quadrupole-time-of-flight mass spectrometry (Q-TOF–MS) was chosen because it can measure accurate masses with high resolution and provides the best information on the molecular composition of the compound of interest, so that its molecular formula can be confirmed, or a preliminary determination made (29, 30). UPLC–Q-TOF–MS analysis revealed the mass of unknown #1 to be *m/z* 244.0498 [M + H]^+^, corresponding to a molecular formula of C_10_H_13_NO_2_S_2_ (Figure 2E). Its main MS/MS fragments were m/z 142.9849 [M + H]^+^ (C_5_H_5_NS_2_^+^), 111.0135 [M + H]^+^ (C_5_H_5_NS^+^), and 79.0436 [M + H]^+^ (C_5_H_5_N^+^). The corresponding neutral losses of 101.0649, 31.9714, and 31.9699 appear to be losses of C_5_H_9_O_2_, S, and S, respectively, which appear to be fragments of the thiol after derivatization.

The derivatization reagent accounts for C_5_H_5_N_*S*_, so the molecular formula of unknown #1 was C_5_H_10_O_2_S. The candidate thiols were screened and identified using the database of flavor molecules (^1,^^2^ accessed in May 2022), then verified by comparison with authentic standard. Unknown #1 was identified as ethyl 2-mercaptopropionate. UPLC–Q-TOF–MS analysis revealed the accurate mass of unknown #2 to be *m/z* 200.0579 [M + H]^+^, corresponding to a molecular formula of C_9_H_14_NS_2_ (Figure 2F). The retention time of unknown #3 was 16.46 min and its molecular ion mass was 214.0736 [M + H]^+^, corresponding to a molecular formula of C_10_H_16_NS_2_ (Figure 2G). Subtracting the formula of the derivatizing reagent gave the molecular formula of unknown #2 and #3 as C_4_H_10_S and C_5_H_12_S, respectively. The database of flavor molecules and comparison with authentic standards unambiguously identified these compounds as 1-butanethiol and 1-pentanethiol, respectively. To our knowledge, this is the first time ethyl 2-mercaptopropionate, 1-butanethiol, and 1-pentanethiol have been detected in Baijiu. Ethyl 2-mercaptopropionate has been reported to be an important odorant, which correlates with age in wine (31). 1-Butanethiol and 1-pentanethiol have burned and sulfuryl odor qualities, which are the odorants of fruit brandy and coffee (32, 33).

### Analytical characteristics of the ultraperformance liquid chromatography–mass spectrometry method

To check the performance and reliability of the newly developed method, quality parameters such as linear range, limit of detection (LOD), limit of quantification (LOQ), precision, and accuracy were determined.

#### Linear range, limits of detection, and limits of quantitation

Linearity was evaluated for each thiol over at least seven different concentrations. The experimentally-determined linear ranges covered a wide concentration range (up to 819 μg/L). The correlation coefficients (*R*^2^) for the thiol derivatives were in the range 0.9911–0.9978. The LOD and LOQ obtained for the thiol derivatives were in the ranges 0.001–0.012 μg/L and 0.003–0.037 μg/L, respectively (Table 2). All LOQs of the thiol derivatives were below their respective odor thresholds (Table 3), making this method suitable for combined chemical analysis and sensory experiments. The UPLC–MS/MS method also performed better than GC for quantification of the volatile thiols (Table 4). For example, the LOQ of 2-furfurylthiol was 0.003 μg/L by UPLC–MS/MS, 200 times lower than by HS-SPME–GC–PFPD (0.60 μg/L) (13). In addition, the UPLC–MS/MS yielded an LOD of 0.001 μg/L for 2-methyl-3-furanthiol, 170 times lower than the 0.17 μg/L achieved by HS-SPME–GC–PFPD (34). Many methods have been proposed for the analysis of thiols in Baijiu, with common techniques involving GC. Several methods such as HS-SPME, SBSE, HS-SPME arrow, and direct injection (DI) coupled with GC are widely used for extraction of thiols in Baijiu. The PFPD, SCD, and TOFMS are highly selective and sensitive for sulfur determination. Compared with previous GC analytical methods for the number of thiols analyzed in Baijiu (Supplementary Figure 2), 11 thiols were studied in the present study [11 vs. 4 with GC × GC–SCD (16), 3 with GC–PFPD (13), 1 with HS-SPME Arrow–GC–MS (18), 0 with LLE–GC × GC–TOFMS (35), 0 with HS-SPME–GC–MS (36), 0 with SBSE–GC–MS (37), and 0 with DI–GC–MS (38)].

**TABLE 2:** Linear range, correlation coefficient (*R*^2^), limits of detection (LOD), and limits of quantitation (LOQ) of the established method for derivatized thiols.

No.	Compounds	Linear equation	Linear range (μg/L)	*R* ^2^	LOD (μg/L)	LOQ (μg/L)
1	Methanethiol	*y* = 0.1028x + 0.0183	0.8–819.2	0.9978	0.012	0.037
2	Ethanethiol	*y* = 0.8996x + 0.4628	0.8–819.2	0.9936	0.008	0.024
3	Ethyl 2-mercaptoacetate	*y* = 4.6146x + 0.1797	0.03–30.72	0.9967	0.002	0.007
4	2-Furfurylthiol	*y* = 0.5528x + 0.1985	0.05–102.4	0.9933	0.001	0.003
5	2-Sulfanylethanol	*y* = 3.5746x + 0.8394	0.01–10.24	0.9950	0.002	0.005
6	2-Methyl-3-furanthiol	*y* = 0.9625x - 0.0310	0.02–10.24	0.9935	0.001	0.003
7	Benzenemethanethiol	*y* = 4.1164x - 0.0676	0.01–10.24	0.9959	0.002	0.007
8	3-Mercaptohexyl acetate	*y* = 4.9971x - 0.0122	0.01–10.24	0.9926	0.002	0.007
9	Ethyl 2-mercaptopropionate	*y* = 9.7719x + 0.2881	0.01–10.24	0.9911	0.002	0.005
10	1-Butanethiol	*y* = 1.3153x - 0.0227	0.01–20.48	0.9929	0.004	0.012
11	1-Pentanthiol	*y* = 1.4120x - 0.6857	0.05–51.2	0.9962	0.007	0.023

**TABLE 3:** The thiol concentrations in different aroma types Baijiu and their corresponding odor activity values (OAVs) range.

No.	Compound	Odor description^a^	Threshold (μg/L)	Concentration (μg/L)				OAV			
				SSAB	RSAB	SAB	LAB	SSAB	RSAB	SAB	LAB
1	Methanethiol	Burnt rubber, gasoline	2.2	229–513	78–245	33–104	2–10	104–233	35–111	15–47	0.9–4.5
2	Ethanethiol	Onion, rubber	0.8	6.7–32.1	5.3–28.4	1.7–7.3	Nd^c^	8.4–40.1	6.6–35.5	2.1–9.1	-
3	Ethyl 2-mercaptoacetate	Cooked vegetable	120	1.3–9.3	0.9–3.3	0.1–0.4	Nd	<0.1	<0.1	<0.1	-
4	2-Furfurylthiol	Coffee, roasted sesame seeds	0.1	11.2–37.8	6.1–21.3	1.7–6.1	0.5–1.9	112–378	61–213	17–61	5–19
5	2-Sulfanylethanol	Garbage, grilled	130	0.03–0.08	0.03–0.07	0.03–0.05	Nd–0.03	<0.1	<0.1	<0.1	<0.1
6	2-Methyl-3-furanthiol	Roasted meat, fried	0.0048	1.0–2.5	0.9–2.8	0.07–0.13	0.08–0.31	208–521	188–583	14.6–27.1	16.7–64.6
7	Benzenemethanethiol	Smoke, roasted	0.01	0.76–3.68	0.09–0.30	0.02–0.05	Nd	76–368	9–30	2–5	-
8	3-Mercaptohexyl acetate	Grapefruit, passion fruit	0.09^b^	0.05–0.13	Nd–0.08	Nd–0.05	Nd	<0.1	<0.1	<0.1	-
9	Ethyl 2-mercaptopropionate	Animal, burnt	13.23^b^	0.64–1.41	0.11–0.34	0.02–0.08	Nd	<0.1	<0.1	<0.1	-
10	1-Butanethiol	Burned, roasted	0.5^b^	1.8–7.6	1.6–8.3	0.07–0.22	Nd	3.6–15.2	3.2–16.6	0.1–0.4	-
11	1-Pentanthiol	Burned, roasted	0.3^b^	1.9–6.2	0.5–3.8	Nd	Nd	6.3–20.7	1.7–12.7	-	-

^a^Odor description are taken from online databases (Flavornet: http://www.flavornet.org; The Good Scents Company: http://www.thegoodscentscompany.com; FlavorDB: https://cosylab.iiitd.edu.in/flavordb/). ^b^Odor threshold detected in this study. ^c^Nd, not detected.

**TABLE 4:** Comparison of analytical methods for thiols in Baijiu.

Compounds	HS-SPME–GC–PFPD^12^		LLE–GC × GC–SCD^16^		HS-SPME Arrow–GC–MS^18^		This study	
				
	LOD	LOQ	LOD	LOQ	LOD	LOQ	LOD	LOQ
Methanethiol	26	86	-	-	-	-	0.012	0.037
Ethanethiol	3.3	11	-	-	-	-	0.008	0.024
2-Furfurylthiol	0.18	0.60	0.12	0.20	0.016	0.053	0.001	0.003
2-Methyl-3-furanthiol	0.17	0.58	0.10	0.21	-	-	0.001	0.003
Benzenemethanethiol	-	-	0.07	0.11	-	-	0.002	0.007

#### Precision and accuracy

The method for precision measurement was based on stable instrument status, and the results were recorded as the intra- and inter-day precision with a number of replicates (*n* = 5) (Table 5). The RSDs of the intra-day measurements varied between 0.31% (1-pentanthiol) and 3.58% (ethyl 2-mercaptoacetate), and the inter-day RSDs varied between 5.24% (ethyl 2-mercaptoacetate) and 11.79% (methanethiol). The accuracy of the method was determined based on the recoveries. The recoveries of the thiol derivatives were all between 81.2% (methanethiol) and 106.7% (1-pentanthiol) at the three different concentration levels studied (low, medium, and high concentrations in the calibration graphs).

**TABLE 5:** Recovery and precision of the established method for derivatized thiols.

No.	Compounds	Spiked level (μg/L)	Recovery (%)	Precision (RSD,%)	
				Intra-day	Inter-day
1	Methanethiol	1	81.2	1.21	7.09
		400	96.8	0.89	8.25
		800	92.5	3.55	11.79
2	Ethanethiol	1	82.5	2.41	6.55
		400	84.6	1.70	8.54
		800	88.5	2.55	11.20
3	Ethyl 2-mercaptoacetate	0.1	88.6	0.63	7.62
		5	92.6	3.21	5.24
		50	97.1	3.58	5.67
4	2-Furfurylthiol	0.1	84.7	1.48	7.89
		10	96.0	1.39	9.31
		100	104.1	1.62	6.79
5	2-Sulfanylethanol	0.02	83.9	2.30	10.28
		0.2	96.5	1.87	6.55
		2	98.3	0.43	7.51
6	2-Methyl-3-furanthiol	0.05	82.5	2.51	9.27
		0.5	104.5	0.97	8.41
		5	96.3	1.88	6.08
7	Benzenemethanethiol	0.05	83.6	3.27	9.50
		0.5	99.4	3.09	7.06
		5	86.7	1.07	7.98
8	3-Mercaptohexyl acetate	0.05	82.2	3.58	5.29
		0.5	83.6	2.96	9.88
		5	96.4	3.06	7.65
9	Ethyl 2-mercaptopropionate	0.02	90.3	0.72	7.35
		0.2	105.4	1.20	8.41
		2	88.4	1.15	8.93
10	1-Butanethiol	0.05	83.0	2.46	6.57
		0.5	81.5	1.86	9.22
		5	96.3	3.20	9.08
11	1-Pentanthiol	0.2	83.9	0.31	7.80
		2	106.7	2.31	6.91
		10	103.6	0.82	9.35

### Analysis of practical samples

#### Quantitation of thiols in Baijiu samples and odor activity value analysis

To determine the practical utility of the new method, it was applied to a variety of typical aroma-type Baijiu samples obtained from diverse regions. The determined concentrations of 11 thiols are shown in Supplementary Table 2. The OAV can be used to evaluate the sensory contribution of an aroma compound, because it is obtained by dividing the concentration of the thiol by its odor threshold. An OAV much greater than 1 means that the compound may contribute to the odor of the Baijiu (39, 40).

The thiol concentrations in different Baijiu aroma types and their corresponding OAV ranges were determined (Table 3). When compared with the other samples, SSAB was characterized by a higher total thiol concentration. The concentrations of seven thiols in SSAB were higher than their corresponding odor thresholds. Methanethiol (229–513 μg/kg, OAVs 104–233), 2-furfurylthiol (11.2–37.8 μg/kg, OAVs 112–378), and 2-methyl-3-furanthiol (1.0–2.5 μg/kg, OAVs 208–521), which have been identified as important aroma compounds for SSAB in previous reports, were also confirmed in this study (34, 41). Two notable thiols, 1-butanethiol (1.8–7.6 μg/L, OAVs 4–15), and 1-pentanethiol (1.9–6.2 μg/L, OAVs 6–21), were quantified in Baijiu for the first time, in trace amounts, but their low odor thresholds still result in significant OAVs.

A total of 7 thiols with OAVs > 1 was found in RSAB. The highest OAV was found for 2-methyl-3-furanthiol (0.9–2.8 μg/kg, OAVs 188–583), which has a roasted meat odor and was identified in RSAB for the first time in this study. 2-Furfurylthiol (6.1–21.3 μg/kg, OAVs 61–213) was found at a concentration much higher than its odor threshold, and determined to be a typical potent odorant of RSAB (12). In the process of RSAB fermentation, *Saccharomyces cerevisiae* generates 2-furfurylthiol using furfural and L-cysteine as precursors (42). The OAVs of ethanethiol (6.6–35.5), benzenemethanethiol (9–30), 1-butanethiol (3.2–16.6), and 1-pentanethiol (1.7–12.7) exceeded their odor thresholds, indicating that they may contribute significantly to the overall aroma profile.

Five thiols were found at concentrations higher than their odor thresholds in SAB. Of these thiols, methanethiol was present at the highest concentration (33–104 μg/kg, OAVs 15–47), followed by ethanethiol (1.7–7.3 μg/kg, OAVs 2–9) and 2-furfurylthiol (1.7–6.1 μg/kg, OAVs 17–61). Lower concentrations were observed for 2-methyl-3-furanthiol (0.07–0.13 μg/kg, OAVs 15–27) and benzenemethanethiol (0.02–0.05 μg/kg, OAVs 2–5), which was present at concentrations below 1 μg/L in all samples. Only four thiols were found in LAB. Among them, methanethiol (2–10 μg/L, OAVs 1–5), 2-furfurylthiol (0.5–1.9 μg/L, OAVs 5–19) and 2-methyl-3-furanthiol (0.08–0.31 μg/L, OAVs 17–65) had OAVs > 1. These findings will facilitate further study of the contribution of thiols to the aroma of Baijiu.

### Statistical analysis of thiols

**FIGURE 3:**
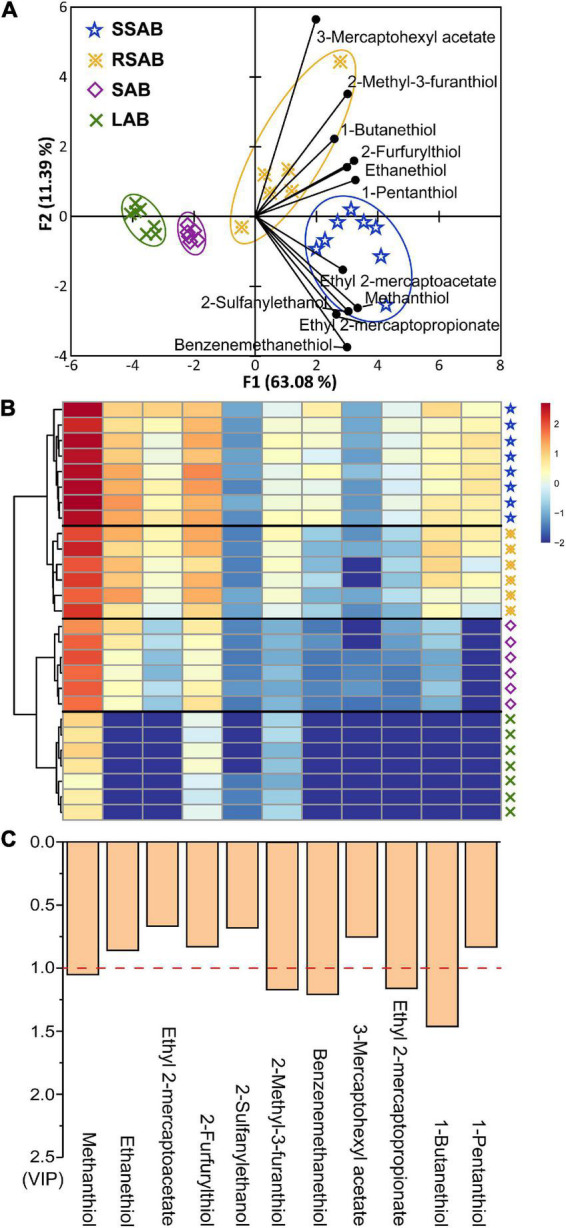
Principal component analysis (PCA) of the thiols in four aroma-types Baijiu samples **(A)**. Heat map of thiols **(B)**. The histogram shows the value of variable importance for projection (VIP) **(C)**.

Principal components analysis (PCA) is an unsupervised method for expressing the similarities and differences of groups of samples (43, 44). PCA analysis was performed on the thiol concentrations (detailed data in Supplementary Table 2) to visualize graphically, the thiol concentrations found in the different Baijiu aroma-types. The PCA scores plot (Figure 3A) clearly distinguished the four different Baijiu aroma-types. The first two principal components PCA (PC1 and PC2) accounted for 74.47% of the variation, indicating that the thiols significantly affect the flavor characteristics of Baijiu (45). Moreover, heat map and hierarchical cluster analysis were also visualized differences within the different Baijiu aroma-types (Figure 3B). The SSAB samples were characterized by the highest thiol concentrations and were all grouped on the far right of the PCA bi-plot. In contrast, the LAB samples with lowest thiol concentrations were on the far left.

To identify the most discriminative thiols contributing to different Baijiu aroma-types, important compounds were calculated (46). Generally, variable importance for projection (VIP) values are considered a significant contributor to different samples. Thiols with VIP > 1.0 are the most relevant for explaining the different Baijiu aroma-types (47). Five thiols (methanethiol, 2-methyl-3-furanthiol, benzenemethanethiol, ethyl 2-mercaptopropionate, and 1-butanethiol) could be considered as being responsible for the differences in the aroma characteristics between Baijiu aroma-types (Figure 3C).

## Conclusion

In this study, a novel strategy using UPLC–MS/MS and UPLC–Q-TOF–MS, was developed to identify thiols in Baijiu. The unique and consistent MS/MS fragmentation pathway of the DTDP-thiol derivatives provides a powerful way to expand the number of quantifiable thiols, and identify unknown thiols by targeting a diagnostic ion (*m/z* 143.5). In addition to the eight known thiols detected in the Baijiu samples, three new thiols were detected and identified (ethyl 2-mercaptopropionate, 1-butanethiol, and 1-pentanethiol) using the strategy developed in this study. In addition, a method using UPLC–MS/MS has been developed and validated to quantify thiols in Baijiu. Seven thiols were suggested as important aroma contributors for Baijiu based on OAVs. Five thiols could be used as markers for different Baijiu aroma-types.

## Data availability statement

The original contributions presented in this study are included in the article/Supplementary material, further inquiries can be directed to the corresponding author.

## Author contributions

YY: conceptualization, methodology, investigation, and writing—original draft. JL, YN, and CL: methodology and resources. SC: conceptualization, writing—review and editing, project administration, and funding acquisition. YX: resources, funding acquisition, and writing—review and editing. All authors have read and approved the final manuscript.

## Footnotes

1
https://cosylab.iiitd.edu.in/flavordb/

2
https://www.vcf-online.nl/VcfHome.cfm
