# Supplementary material for: Characterization of volatile thiols in Chinese liquor (Baijiu) by ultraperformance liquid chromatography–mass spectrometry and ultraperformance liquid chromatography–quadrupole-time-of-flight mass spectrometry
Source: Front Nutr. 2022 Oct 3;9:1022600. doi: 10.3389/fnut.2022.1022600 (PMC9574356; doi:10.3389/fnut.2022.1022600)
Supplement: Supplementary file 1 [file Data_Sheet_1.docx]

Supplementary Material

**Table Supplementary 1.** Information of Baijiu samples in this study

| No. | Type | Name | Abbreviation | Manufacturer | Location | Alcohol % by Volume |
| --- | --- | --- | --- | --- | --- | --- |
| 1 | Soy sauce aroma-type Baijiu (SSAB) | GUO TAI GUO BIAO | GT | GUI ZHOU GUOTAI LIQUOR CO., LTD. | Beijing,  China | 53 |
| 2 |  | MOUTAI PRINCE | MT | KWEICHOW MOUNTAI CO., LTD. | Zunyi,  China | 53 |
| 3 |  | ZHAI YAO | ZY | GUI ZHOU JINSHAJIAO LIQUOR CO., LTD. | Zunyi,  China | 53 |
| 4 |  | QIN HAN ZHANG JIU | QHZ | KWEICHOW MOUNTAI QINHANZHANG JIUYE CO., LTD. | Zunyi,  China | 53 |
| 5 |  | DIAOYUTAI AMBASSADOR LIQUOR | DYT | KWEICHOW DIAOYUTAI STATE GUEST DISTILLERY CO., LTD. | Zunyi,  China | 53 |
| 6 |  | JIAO CANG | XJ | GUIZHOU MAOTAI OISTILLERY(GROUP) XI JIU CO., LTD. | Zunyi,  China | 53 |
| 7 |  | LANG PAI LANG JIU | LJ | SICHUAN GULIN LANGJIU DISTILLERY CO., LTD. | Luzhou,  China | 53 |
| 8 |  | ZHEN WU | ZJ | GUIZHOU ZHENJIU LIQUOR-MAKING CO., LTD. | Zunyi,  China | 53 |
| 1 | Roasted sesame-like aroma-type Baijiu  (RSAB) | YIPINJINGGZHI MIAOPIN | JZ-1 | SHANDONG JINGZHI LIQUOR CO., LTD. | Weifang,  China | 46 |
| 2 |  | YIPINJINGGZHI JIPIN | JZ-2 | SHANDONG JINGZHI LIQUOR CO., LTD. | Weifang,  China | 42 |
| 3 |  | GUO JING G9 | BDJ-1 | SHANDONG BANDAOJING CO., LTD. | Zibo,  China | 52 |
| 4 |  | GUO JING ZUN PIN | BDJ-2 | SHANDONG BANDAOJING CO., LTD. | Zibo,  China | 52 |
| 5 |  | GUO JING HONG SE JING DIAN | BDJ-3 | SHANDONG BANDAOJING CO., LTD. | Zibo,  China | 46 |
| 6 |  | MEILANFANG | MLF | TAIZHOU MEILANCHUN LIQUOR CO., LTD. | Taizhou,  China | 53 |
| 1 | Strong aroma-type Baijiu  (SAB) | WULIANGYE JIAOBEI | WLY-1 | WULIANGYE YIBIN CO., LTD. | Yibin,  China | 52 |
| 2 |  | WULIANGYE 1618 | WLY-2 | WULIANGYE YIBIN CO., LTD. | Yibin,  China | 52 |
| 3 |  | LUZHOULAOJIAO TEQV | LZLJ | LUZHOU LAOJIAO CO., LTD. | Luzhou,  China | 52 |
| 4 |  | JIN JIAN NAN CHIEW K6 | JNC-1 | MIANZHU JIANNANCHUN DISTILLERY CO., LTD. | Mianzhu,  China | 52 |
| 5 |  | JIANNANCHUN | JNC-2 | MIANZHU JIANNANCHUN DISTILLERY CO., LTD. | Mianzhu,  China | 52 |
| 6 |  | QUANXINGDAQV QINGHUA | QXDQ | SICHUN QUANXING DISTILLERY CO., LTD. | Chengdu,  China | 52 |
| 1 | Light aroma-type Baijiu  (LAB) | HONGXINGERGUOTOU QINGHUACI | HX-1 | BEIJING RED STAR CO.,LTD. | Beijing,  China | 52 |
| 2 |  | HONGXINGERGUOTOU NIANFENYUANJIANG 10 | HX-2 | BEIJING RED STAR CO.,LTD. | Beijing,  China | 46 |
| 3 |  | FENJIU HUANGGAI | FJ | SHANXI XINGHUACUN FENJIU DISTILLERY CO., LTD. | Fenyang,  China | 53 |
| 4 |  | NIULANSHAN ERGUOTOU JINGDIAN | NLS-1 | BEIJING SHUNXIN AGRICULTURE CO.,LTD | Beijing,  China | 52 |
| 5 |  | NIULANSHAN ERGUOTOU ZHENPIN | NLS-2 | BEIJING SHUNXIN AGRICULTURE CO.,LTD | Beijing,  China | 45 |
| 6 |  | TIANYOUDE GUOZHIDE | TYD-1 | QINGHAI HUZHU HIGHLAND BARLEY WINE CO.,LTD. | Haidong,  China | 52 |
| 7 |  | TIANYOUDE QICAIHUZHU | TYD-2 | QINGHAI HUZHU HIGHLAND BARLEY WINE CO.,LTD. | Haidong,  China | 42 |

**Table Supplementary 2.** Concentrations (μg/L) of thiols in Baijiu samples

|  | Methan  ethiol | Ethan  ethiol | Ethyl 2-merca  ptoacetate | 2-Furfuryl  thiol | 2-Sulfanyl  ethanol | 2-Methyl-3-furanthiol | Benzeneme  thanethiol | 3-Mercaptohexyl acetate | Ethyl 2-mercapto  propionate | 1-Butan  ethiol | 1-Pentan  ethiol |
| --- | --- | --- | --- | --- | --- | --- | --- | --- | --- | --- | --- |
| GT | 462±3.0 | 9.2±0.6 | 9.32±0.2 | 11.2±0.6 | 0.079±2.1 | 0.95±0.3 | 3.68±1.2 | 0.081±3.5 | 0.876±3.7 | 7.6±1.7 | 1.9±2.1 |
| MT | 407±1.2 | 11.2±0.6 | 1.25±2.2 | 22.4±1.5 | 0.042±3.2 | 2.48±1.1 | 1.71±0.6 | 0.089±4.6 | 0.925±4.5 | 3.2±0.6 | 6.2±0.1 |
| ZY | 513±1.7 | 32.1±1.5 | 2.75±0.3 | 18.5±0.9 | 0.082±2.9 | 1.46±1.0 | 1.64±0.6 | 0.051±2.8 | 1.132±3.6 | 4.1±0.9 | 3.9±0.4 |
| QHZ | 379±1.1 | 21.5±1.2 | 4.90±0.6 | 21.3±1.3 | 0.041±3.0 | 2.53±0.9 | 2.21±0.7 | 0.063±3.1 | 0.750±3.0 | 4.6±1.2 | 3.5±0.9 |
| DYT | 362±1.1 | 22.8±2.1 | 1.82±0.9 | 37.8±2.5 | 0.062±1.9 | 1.09±1.7 | 2.53±1.9 | 0.128±2.0 | 0.823±2.8 | 3.0±0.8 | 5.6±1.5 |
| XJ | 421±0.9 | 19.3±1.0 | 3.49±0.9 | 28.4±2.8 | 0.033±3.5 | 1.12±1.6 | 1.05±1.1 | 0.054±2.9 | 0.642±3.7 | 2.1±1.5 | 5.4±0.3 |
| LJ | 337±1.7 | 10.2±3.3 | 1.26±1.8 | 12.2±0. 9 | 0.069±2.7 | 2.03±0.4 | 0.76±2.0 | 0.059±3.9 | 1.367±1.4 | 2.5±1.9 | 4.0±2.1 |
| ZJ | 229±0.8 | 6.7±2.2 | 2.71±2.0 | 16.7±1.3 | 0.051±2.2 | 1.53±0.9 | 1.53±1.0 | 0.073±4.4 | 1.414±2.5 | 1.8±2.3 | 3.2±2.0 |
| JZ-1 | 78±3.1 | 28.4±1.7 | 1.65±2.8 | 19.3±1.0 | 0.072±1.9 | 1.71±0.5 | 0.091±1.9 | 0.074±4.1 | 0.335±1.7 | 1.6±0.7 | 1.4±1.7 |
| JZ-2 | 157±2.9 | 5.3±1.6 | 0.89±3.5 | 8.5±0.5 | 0.065±2.0 | 0.93±0.5 | 0.225±2.3 | 0.041±3.0 | 0.109±2.9 | 2.5±1.1 | 0.5±1.5 |
| BDJ-1 | 97±4.3 | 14.3±2.7 | 1.81±2.2 | 6.1±0.1 | 0.028±2.8 | 2.05±1.7 | 0.118±1.9 | Nd | 0.275±3.70 | 8.2±1.0 | 0.7±2.3 |
| BDJ-2 | 113±1.3 | 19.2±1.2 | 3.06±3.2 | 21.3±1.1 | 0.030±3.9 | 2.76±2.1 | 0.183±2.5 | 0.043±2.7 | 0.262±4.1 | 6.0±2.3 | 3.8±2.8 |
| BDJ-3 | 142±1.1 | 11.1±3.2 | 1.70±0.9 | 17.5±0.9 | 0.031±2.2 | 1.39±0.9 | 0.302±2.9 | Nd | 0.154±4.4 | 6.4±1.7 | 2.7±1.0 |
| MLF | 245±2.5 | 7.2±2.8 | 3.27±0.8 | 18.2±1.5 | 0.034±2.7 | 1.52±0.3 | 0.103±3.1 | 0.082±3.6 | 0.136±1.4 | 8.3±2.5 | 3.1±2.8 |
| WLY-1 | 83±1.2 | 2.5±2.9 | 0.35±4.1 | 3.5±3.1 | 0.049±1.8 | 0.07±1.5 | 0.033±2.5 | 0.049±3.7 | 0.026±2.9 | 0.17±1.6 | Nd |
| WLY-2 | 59±0.7 | 1.9±3.0 | 0.17±3.2 | 6.1±2.5 | 0.027±1.5 | 0.11±0.7 | 0.031±3.3 | 0.021±2.9 | 0.030±1.8 | 0.10±2.9 | Nd |
| LZLJ | 104±1.1 | 2.1±2.1 | 0.14±0.4 | 1.8±3.3 | 0.025±2.6 | 0.08±2.1 | 0.027±2.9 | 0.028±3.0 | 0.037±2.8 | 0.07±2.8 | Nd |
| JNC-1 | 33±3.0 | 7.3±2.9 | 0.22±0.1 | 4.1±1.0 | 0.032±3.7 | 0.09±1.1 | 0.025±3.0 | Nd | 0.079±3.5 | 0.22±0.5 | Nd |
| JNC-2 | 76±2.6 | 4.3±2.4 | 0.35±0.7 | 2.2±0.4 | 0.028±3.9 | 0.11±3.1 | 0.047±2.8 | Nd | 0.061±1.4 | 0.19±2.0 | Nd |
| QXDQ | 69±2.9 | 1.7±3.7 | 0.11±2.5 | 1.7±3.1 | 0.031±4.0 | 0.13±2.7 | 0.021±2.5 | 0.033±2.7 | 0.024±2.0 | 0.08±0.9 | Nd |
| TYD-1 | 9.6±0.5 | Nd | Nd | 1.49±2.2 | Nd | 0.11±2.2 | Nd | Nd | Nd | Nd | Nd |
| TYD-2 | 8.1±0.4 | Nd | Nd | 1.05±3.5 | Nd | 0.24±1.9 | Nd | Nd | Nd | Nd | Nd |
| NLS-1 | 3.7±2.3 | Nd | Nd | 0.54±5.1 | 0.031±2.9 | 0.19±1.3 | Nd | Nd | Nd | Nd | Nd |
| NLS-2 | 5.1±1.2 | Nd | Nd | 0.69±2.5 | Nd | 0.31±0.1 | Nd | Nd | Nd | Nd | Nd |
| HX-1 | 5.3±2.0 | Nd | Nd | 1.87±2.3 | Nd | 0.15±1.4 | Nd | Nd | Nd | Nd | Nd |
| HX-2 | 4.9±0.1 | Nd | Nd | 0.92±3.1 | 0.022±3.7 | 0.27±0.3 | Nd | Nd | Nd | Nd | Nd |
| FJ | 2.1±3.1 | Nd | Nd | 0.71±0.6 | 0.027±3.5 | 0.08±0.9 | Nd | Nd | Nd | Nd | Nd |

Nd: not detected.

Values expressed as mean ± relative standard deviation for three determinations.


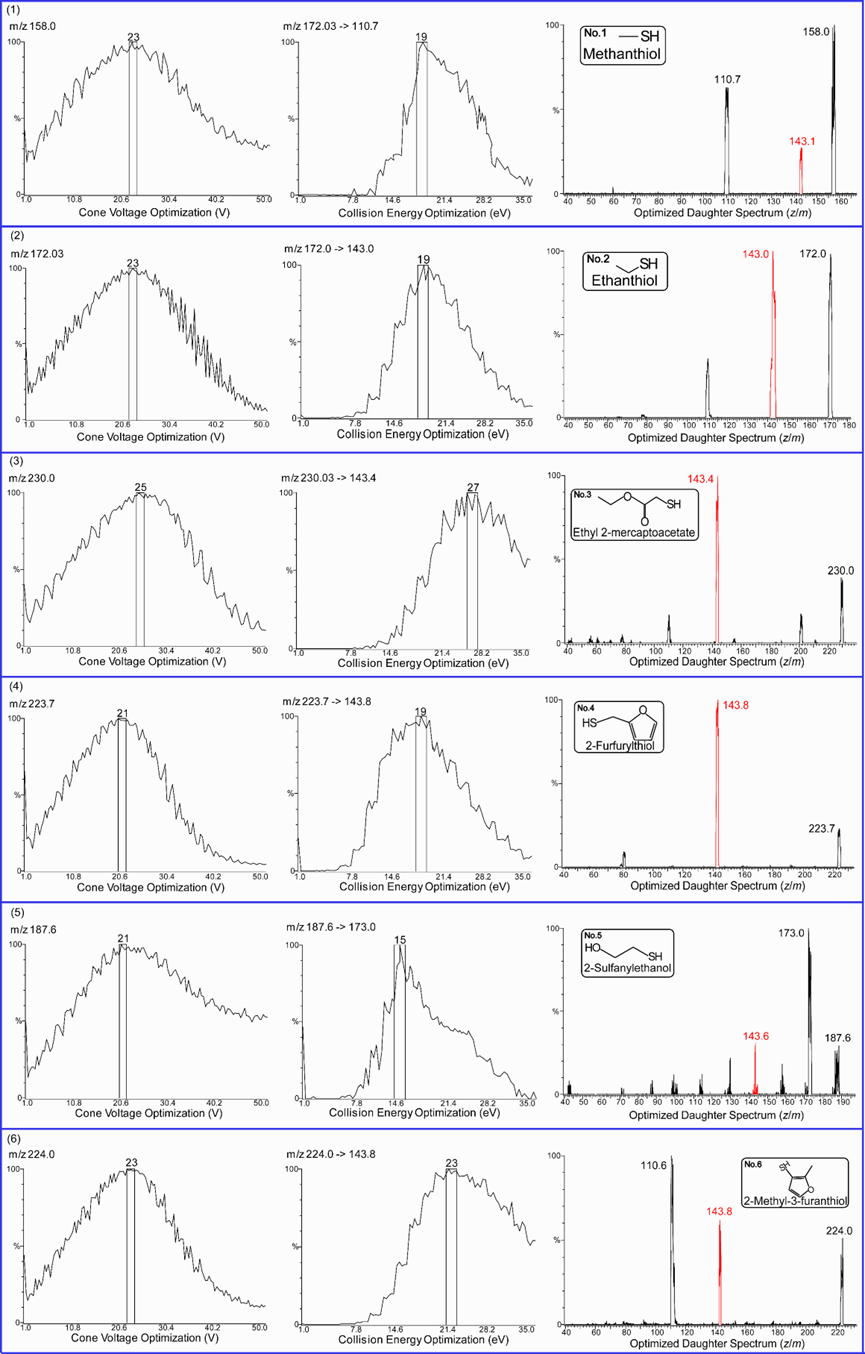


**Supplementary Figure 1**. MS/MS method optimization and mass spectra for (1) methanethiol, (2) ethanethiol, (3) ethyl 2-mercaptoacetate, (4) 2-furfurylthiol, (5) 2-sulfanylethanol, and (6) 2-methyl-3-furanthiol.


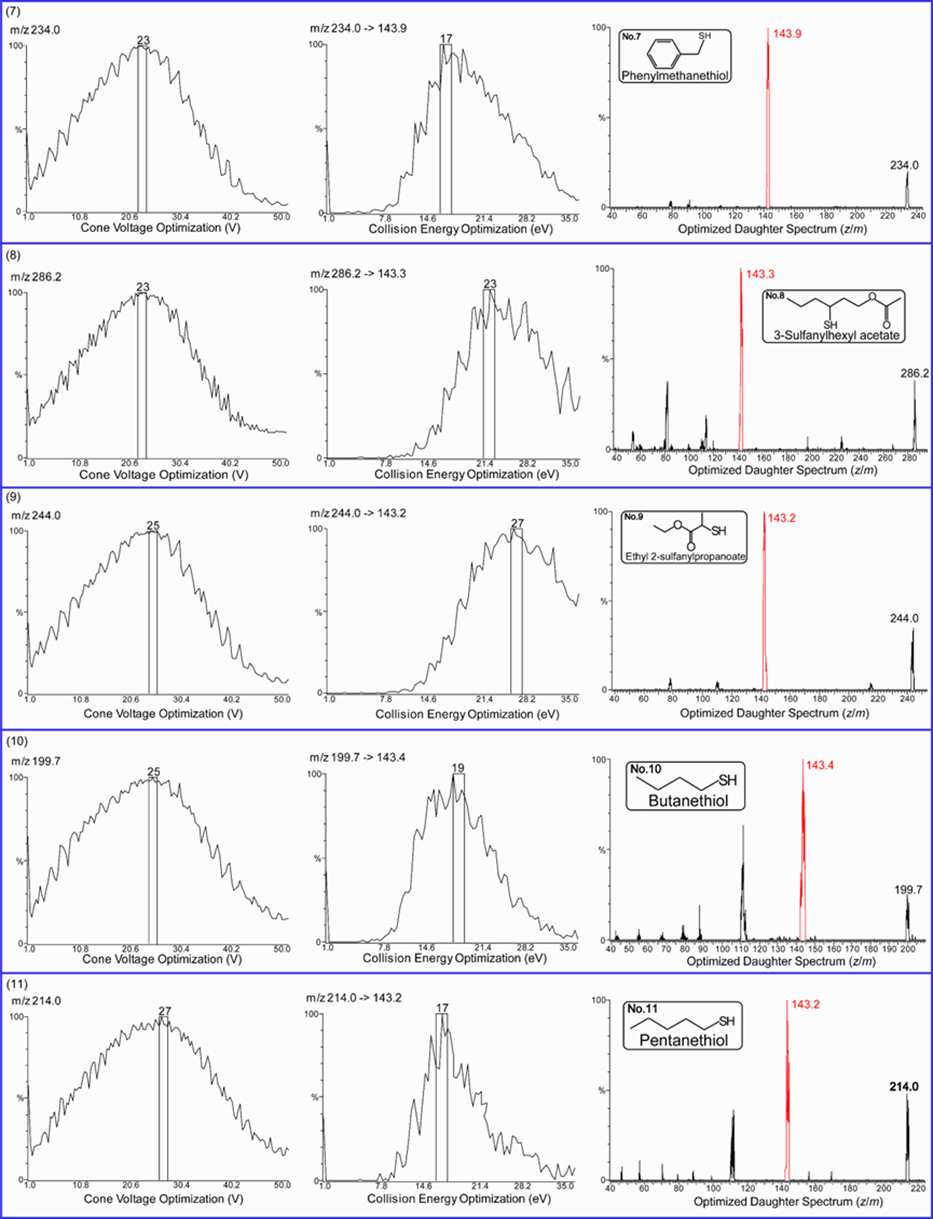


**Supplementary Figure 1** contd. MS/MS method optimization and mass spectra for (7) benzenemethanethiol, (8) 3-mercaptohexyl acetate, (9) ethyl 2-mercaptopropionate, (10) 1-butanethiol, (11) 1-pentanthiol


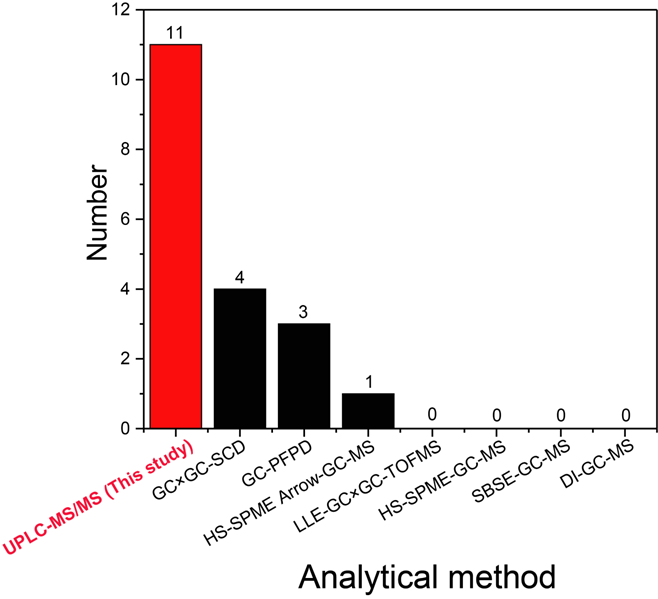


**Supplementary Figure 2**. Comparison of quantitative thiols obtained by different analytical methods in Baijiu (1-7)

**References**

1. Song X, Zhu L, Wang X, Zheng F, Zhao M, Liu Y, Li H, Zhang F, Zhang Y, Chen F. Characterization of key aroma-active sulfur-containing compounds in Chinese Laobaigan Baijiu by gas chromatography-olfactometry and comprehensive two-dimensional gas chromatography coupled with sulfur chemiluminescence detection. *Food Chem*. (2019) 297:124959. doi:10.1016/j.foodchem.2019.124959.

2. Chen S, Sha S, Qian M, Xu Y. Characterization of volatile sulfur compounds in Moutai liquors by headspace solid-phase microextraction gas chromatography-pulsed flame photometric detection and odor activity value. *J Food Sci*. (2017) 82:2816-2822. doi:10.1111/1750-3841.13969.

3. Zhang X, Wang C, Wang L, Chen S, Xu Y. Optimization and validation of a head space solid-phase microextraction-arrow gas chromatography-mass spectrometry method using central composite design for determination of aroma compounds in Chinese liquor (Baijiu). *J Chromatogr A*. (2019):460584. doi:10.1016/j.chroma.2019.460584.

4. Zhu S, Lu X, Ji K, Guo K, Li Y, Wu C, Xu G. Characterization of flavor compounds in Chinese liquor Moutai by comprehensive two-dimensional gas chromatography/time-of-flight mass spectrometry. *Anal Chim Acta*. (2007) 597:340–348. doi:10.1016/j.aca.2007.07.007.

5. Ding X, Wu C, Huang J, Zhou R. Characterization of interphase volatile compounds in Chinese Luzhou-flavor liquor fermentation cellar analyzed by head space-solid phase micro extraction coupled with gas chromatography mass spectrometry (HS-SPME/GC/MS). *LWT - Food Sci Tec*. (2016) 66:124-133. doi:10.1016/j.lwt.2015.10.024.

6. Fan W, Shen H, Xu Y. Quantification of volatile compounds in Chinese soy sauce aroma type liquor by stir bar sorptive extraction and gas chromatography-mass spectrometry. *J Sci Food Agric*. (2011) 91:1187-1198. doi:10.1002/jsfa.4294.

7. Sun J, Zhao D, Zhang F, Sun B, Zheng F, Huang M, Sun X, Li H. Joint direct injection and GC–MS chemometric approach for chemical profile and sulfur compounds of sesame-flavor Chinese Baijiu (Chinese liquor). *Eur Food Res Technol*. (2017) 244:145-160. doi:10.1007/s00217-017-2938-7.
